# Association of oxidative balance score, cardiovascular, and all-cause mortality among patients with type 2 diabetes mellitus

**DOI:** 10.3389/fendo.2024.1429662

**Published:** 2024-08-20

**Authors:** Chengming Ni, Xiaohang Wang, Yunting Zhou, Qianqian Wang, Zhensheng Cai, Huan Wang, Yang Chen, Yu Liu, Zilin Sun

**Affiliations:** ^1^ Department of Endocrinology, Zhongda Hospital, Institute of Diabetes, School of Medicine, Southeast University, Nanjing, China; ^2^ Institute of Diabetes, School of Medicine, Southeast University, Nanjing, China; ^3^ Department of Endocrinology, The Affiliated Hospital of Yangzhou University, Yangzhou University, Yangzhou, China; ^4^ Department of Endocrinology, Nanjing First Hospital, Nanjing Medical University, Nanjing, Jiangsu, China

**Keywords:** oxidative balance score, cardiovascular, all-cause mortality, type 2 diabetes mellitus, dietary

## Abstract

**Background:**

To investigate the association between oxidative balance score (OBS), cardiovascular mortality (CVM), and all-cause mortality (ACM) in type 2 diabetes mellitus (T2DM) patients.

**Methods:**

We included 6,119 participants with T2DM from the 2005-2020 National Health and Nutrition Examination Surveys (NHANES). The status of CVM and ACM of participants was followed through December 31, 2019. Multivariable Cox regression models, Kaplan-Meier curves, log-rank test, restricted cubic spline regression, and subgroup analysis, were used to evaluate the relationship between OBS, CVM, and ACM.

**Results:**

During a median of 100.9 months follow-up, 1,790 ACM cases had occurred, 508 of which were due to cardiovascular disease. The T2DM participants were divided into four groups based on the quartiles of OBS. Participants with Q4 tended to be younger, financially better-off, married, highly educated, had lower alcohol consumption rates, were non-smokers, and exhibited a lower likelihood of ACM and CVM. In multivariate Cox regression models, compared with the patients with Q4, those with Q1 had a 30% increased risk for ACM (Q1, reference; Q4, HR: 0.70, 95%CI: 0.58-0.86) and a 43% increased risk for CVM (Q1, reference; Q4, HR: 0.57, 95%CI: 0.36-0.88). The restricted cubic spline regression models have no nonlinear relationship between OBS, CVM, and ACM. Kaplan-Meier survival curves showed that patients with Q4 had a lower risk of ACM and CVM (log-rank P < 0.05).

**Conclusions:**

We find that ACM and CVM increase with higher OBS in T2DM patients. Moreover, there are linear relationships between OBS, ACM, and CVM.

## 1 Introduction

Type 2 diabetes mellitus (T2DM), a chronic metabolic disorder, has exhibited a significant growth trend globally due to changes in lifestyle, population aging, and accelerated urbanization. The number of adults with diabetes has increased from 108 million in 1980 to 463 million in 2019, and 90-95% of these cases are T2DM patients (1). It is predicted that the prevalence of T2DM among adults in the United States will reach 25-28% by 2050 (2). T2DM not only has a profound impact on patients’ quality of life but also can trigger a series of severe complications, posing a significant threat to patients’ health and life. This has become one of the significant challenges in the current public health landscape. Diabetes is a cause of one in nine deaths among adults aged 20-79, emphasizing the crucial clinical significance of identifying an appropriate predictor for T2DM mortality (3).

Cardiovascular disease is the principal cause of death and morbidity among T2DM patients (4). According to statistics, the risk of cardiovascular disease in T2DM patients is twice that of non-diabetic patients. Atherosclerosis and heart failure are the most common complications in T2DM patients, and they are significant contributors to morbidity and mortality (5). Current research indicates that circulating metabolites such as hexanoylcarnitine, tryptophan, and kynurenine have been confirmed to be associated with and improve the prediction of all-cause mortality (ACM) in T2DM patients (6). Furthermore, high uric acid levels in T2DM are linked to cardiovascular mortality (CVM). However, while these biomarkers have improved prediction accuracy to a certain extent, their popularity and clinical application value still require further validation.

The oxidative balance score (OBS) is a composite index based on quantiles or categories related to dietary/lifestyle exposures (7). The OBS measures the balance between oxidant and antioxidant exposures, with a higher OBS indicating a dominance of antioxidant exposure, studies have shown a correlation between OBS and the occurrence of T2DM and cardiovascular diseases (8, 9). OBS has been found to have a significant correlation with the incidence of non-alcoholic fatty liver disease (NAFLD) and metabolic dysfunction-associated fatty liver disease (MASLD) among American adults, as well as with ACM associated with MASLD (10, 11). In addition, OBS has been associated with depression, sleep quality, periodontitis, and kidney disease (12–15). Another study has demonstrated that OBS is inversely associated with ACM and CVM (16). By measuring OBS, we can gain a deeper understanding of the pathogenesis of related diseases, providing novel insights into their treatment and prevention.

OBS possesses a unique advantage in assessing the prevalent oxidative stress state in T2DM. However, the current understanding of the relationship between OBS and ACM or CVM among T2DM is still unclear. Therefore, conducting in-depth studies to explore the association between OBS and the prognosis of T2DM is of significant importance and value to the research.

## 2 Method

### 2.1 Study population

**Figure 1:**
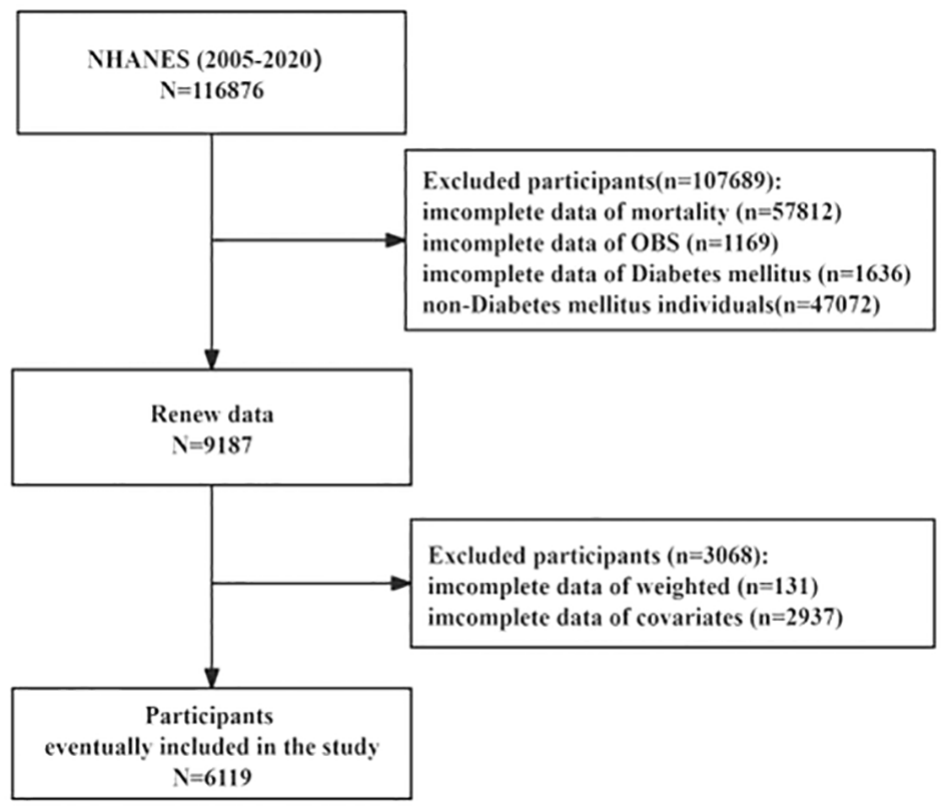
Flow chart of the sample selection from NHANES 2007-2016.

The National Health and Nutrition Examination Surveys (NHANES), a program sponsored by the Centers for Disease Control and Prevention (CDC), aims to evaluate the American populace’s health status comprehensively. The data utilized for our analyses were drawn from the NHANES database spanning 2005 to 2020, encompassing 116,876 participants. The Institutional Review Board of the Centers for Disease Control and Prevention approved the NHANES study protocol, ensuring rigorous ethical standards were upheld. In this study, non-T2DM participants without complete information on mortality, OBS, T2DM, weighted, and covariates were excluded; we enrolled 6,119 participants for the final analysis of this research (**Figure 1**). The survival status of participants was followed up to December 31, 2019. Additionally, all participants gave informed consent, indicating their voluntary participation and understanding of the study’s objectives and procedures.

### 2.2 Exposure definitions

The calculation of OBS is based on early research (13, 17, 18). OBS was divided into 16 dietary OBS and four lifestyle OBS, including two prooxidants and 14 antioxidants (19).

The dietary factors were classified into prooxidants (total fat and iron) and antioxidants (β-carotene, dietary fiber, copper, vitamin B6, vitamin B12, vitamin C, niacin, vitamin E, total folate, vitamin B2, magnesium, calcium, zinc, and selenium) according to the effect on oxidative stress. Lifestyle factors were classified as prooxidants (alcohol intake, BMI, and cotinine) and antioxidants (physical activity). Dietary OBS components were assessed in the NHANES using 24-hour food recalls. Physical activity, expressed as weekly metabolic equivalents (MET), was calculated using data on leisure time activities over the past 30 days acquired from household interviews. The primary exposure was OBS and the primary outcomes were ACM and CVM. We used multivariate Cox regression models to assess these relationships, with stepwise adjustments to control for potential confounders.

### 2.3 Data collection

Questionnaires were collected at baseline to obtain demographic information, including age, personal income ratio (PIR), body mass index (BMI), sex, race, marital status, and education level. Additionally, personal medical history was assessed for T2DM, hypertension, and cardiovascular disease (CVD). Smoking status was categorized as former, current, or never while drinking status was classified as no, moderate, or heavy.

Physical examinations included height, weight, systolic blood pressure (SBP), and diastolic blood pressure (DBP). Blood samples were collected after an 8-hour fast to measure fasting blood glucose (FBG) and conduct other laboratory tests.

### 2.4 Clinical outcome

ACM refers to deaths occurring for any reason, including cardiovascular disease or cerebrovascular disease, prior to December 31, 2019. Mortality data were obtained from the NHANES-linked mortality files covering 1999-2019. We censored the time from enrollment (date of interview) to death. CVM was defined using the International Classification of Diseases, Tenth Revision codes (I00-I09, I11, I13, I20-I51). Participants who did not have any recorded deaths during the follow-up period were considered alive.

### 2.5 Statistical analysis

The data were processed by NHANES analytical guidelines (20–22). Continuous variables were expressed as mean ± standard deviation for normally distributed variables or median (interquartile range) if the data were not normally distributed. Categorical variables were presented as numbers (n) and percentages (%). The one-way ANOVA (continuous variables with Gaussian distribution), Kruskal-Wallis H-test (continuous variables with non-Gaussian distribution), or chi-square tests (Differences between groups for categorical variables) were used to assess differences according to OBS quartiles (Q1 ≤ 13, 13<Q2 ≤ 18, 18<Q3 ≤ 24, Q4>24) in groups. Multivariable Cox regression analysis was used to estimate the adjusted hazard ratio (HR) and 95% confidence interval (95% CI) for ACM and CVM according to OBS, lifestyle OBS, and dietary OBS. We used a multivariate Cox regression model to estimate adjusted HRs and 95% CIs because Cox proportional risk models are suitable for analyzing survival data and can handle multiple covariates. Model 1 was constructed without adjusted covariates. Model 2 adjusts for age, sex, and race. Model 3 further adjusts PIR, marital status, education level, smoking status, and hypertension. We performed survival analysis using standardized Kaplan-Meier curves and the log-rank test. The restricted cubic spline (RCS) regression model then tested the association between OBS, ACM, and CVM. Finally, we conducted a subgroup analysis, including age (<60 or ≥60 years), sex (male or female), BMI (<30 or ≥30 kg/m^2^), smoking (former, now or never), and hypertension (yes or no).

Statistical significance was defined as a two-sided P-value < 0.05. R version 3.3.2 (R Foundation for Statistical Computing, Vienna, Austria) was used for all statistical analysis.

## 3 Results

### 3.1 Baseline characteristics

During the continuous NHANES cycles from 2005 to 2020, a total of 6,119 participants were included. **Table 1** presents the baseline characteristics of the study participants, categorized by quartiles of OBS. The average age of the included participants was 59.06 ± 0.27 years, with 3,012 females (49.43%). Among the participants, there were 1,198 Mexican Americans (8.44%), 1,522 Non-Hispanic Black individuals (13.89%), 2,452 Non-Hispanic White individuals (66.20%), 526 Other Hispanic individuals (5.13%), and 421 other race individuals (6.34%). While no significant differences were observed in sex, BMI and C-reactive protein (CRP) across the quartiles of OBS, significant variations were found among participants in different quartiles concerning age, PIR, race, marital status, education level, alcohol consumption, smoking, hypertension, and CVD. Specifically, the prevalence of CVD from the lowest to the highest quartile of the OBS was 31.99%, 24.09%, 21.76%, and 19.72%, respectively. In addition, participants with Q4 tended to be younger, financially better-off, married, highly educated, have lower alcohol consumption rates, be non-smokers, and exhibit a lower likelihood of ACM and CVM. Besides, compared to deceased patients, survivors show higher OBS, dietary OBS, and lifestyle OBS. More information can be found in **Table 2**.

**Table 1:** Baseline characteristics of participants according to the OBS quartiles.

	Overall	OBS quartiles				P value
		Q1	Q2	Q3	Q4	
Age, years	59.06(0.27)	59.78(0.46)	59.78(0.54)	58.91(0.45)	58.03(0.47)	0.015
Sex, n (%)						0.647
Female	3012(49.93)	788(47.98)	723(51.24)	821(50.44)	680(50.04)	
Male	3107(50.07)	981(52.02)	695(48.76)	780(49.56)	651(49.96)	
Race, n (%)						<0.001
Mexican American	1198(8.44)	311(7.50)	287(8.83)	322(8.97)	278(8.39)	
Non-Hispanic Black	1522(13.89)	591(21.24)	361(15.77)	335(11.38)	235(8.69)	
Non-Hispanic White	2452(66.20)	645(60.40)	541(64.54)	668(66.64)	598(72.08)	
Other Hispanic	526(5.13)	133(5.80)	132(4.84)	151(5.72)	110(4.16)	
Other race	421(6.34)	89(5.06)	97(6.02)	125(7.29)	110(6.69)	
PIR	2.82(0.04)	2.31(0.06)	2.63(0.06)	2.96(0.06)	3.26(0.07)	<0.001
Marital status, n (%)						0.003
Separated	1901(26.42)	585(30.28)	460(26.46)	498(27.36)	358(22.07)	
Married	3696(65.19)	1016(59.57)	846(65.43)	960(64.70)	874(70.34)	
Never married	522(8.39)	168(10.15)	112(8.11)	143(7.93)	99(7.59)	
Education level, n (%)						<0.001
Below high school	1049(8.59)	393(13.52)	274(9.92)	248(7.61)	134(4.31)	
High school	2514(39.38)	816(48.01)	592(41.60)	610(36.47)	496(33.21)	
Above high school	2556(52.03)	560(38.47)	552(48.48)	743(55.92)	701(62.47)	
Drinking status, n (%)						<0.001
No	2840(39.15)	923(46.22)	683(42.27)	700(35.85)	534(33.99)	
Moderate	2539(48.36)	627(40.94)	566(44.42)	712(50.77)	634(55.39)	
Heavy	740(12.50)	219(12.84)	169(13.30)	189(13.38)	163(10.62)	
BMI, kg/m^2^	33.20(0.15)	33.38(0.27)	33.10(0.25)	33.17(0.30)	33.14(0.30)	0.880
CRP	0.61(0.03)	0.74(0.06)	0.60(0.04)	0.56(0.03)	0.54(0.05)	0.050
Smoking status, n (%)						<0.001
Former	2133(35.42)	616(33.22)	505(37.07)	569(35.44)	443(35.97)	
Never	2990(48.27)	743(39.76)	684(46.80)	808(50.07)	755(54.90)	
Now	996(16.30)	410(27.01)	229(16.13)	224(14.49)	133(9.13)	
Hypertension, n (%)						0.017
No	1695(29.25)	432(25.61)	381(27.40)	459(30.36)	423(32.72)	
Yes	4424(70.75)	1337(74.39)	1037(72.60)	1142(69.64)	908(67.28)	
CVD						<0.001
No	4534(75.91)	1188(68.01)	1062(75.91)	1219(78.24)	1065(80.28)	
Yes	1585(24.08)	582(31.99)	355(24.09)	382(21.76)	266(19.72)	
ACM, n (%)						<0.001
No	4329(78.76)	1107(69.60)	1004(78.90)	1169(80.06)	1049(85.17)	
Yes	1790(21.24)	662(30.40)	414(21.10)	432(19.94)	282(14.83)	
CVM, n (%)						<0.001
No	5611(93.76)	1570(90.68)	1301(93.86)	1476(93.82)	1264(96.27)	
Yes	508(6.24)	199(9.32)	117(6.14)	125(6.18)	67(3.73)	

OBS, oxidative balance score; CVM, cardiovascular mortality; ACM, all-cause mortality; PIR, poverty income ratio; BMI, body mass index; CRP, C-reactive protein; CVD, cardiovascular disease.

OBS quartiles: Q1 ≤ 13, 13< Q2 ≤ 18, 18 < Q3 ≤ 24, and Q4 > 24.

All values are expressed as a weighted proportion (%) or mean ± standard error.

**Table 2:** Characteristics of the study population grouped by survival status.

	Overall	survival	non-survival	P value
Age, years	59.06(0.27)	56.59(0.29)	68.21(0.38)	<0.001
Sex, n (%)				0.144
Female	3012(49.94)	2213(50.52)	799(47.77)	
Male	3107(50.07)	2116(49.48)	991(52.23)	
Race, n (%)				<0.001
Mexican American	1198(8.44)	915(9.39)	283(4.94)	
Non-Hispanic Black	1522(13.89)	1104(14.21)	418(12.72)	
Non-Hispanic White	2452(66.20)	1505(63.73)	947(75.36)	
Other Hispanic	526(5.13)	439(5.72)	87(2.93)	
Other race	421(6.34)	366(6.95)	55(4.05)	
PIR	2.821(0.04)	2.946(0.04)	2.357(0.06)	<0.001
Marital status, n (%)				<0.001
Separated	1901(26.42)	1154(22.89)	747(39.51)	
Married	3696(65.19)	2758(68.07)	938(54.53)	
Never married	522(8.39)	417(9.05)	105(5.96)	
Education level, n (%)				<0.001
Below high school	1049(8.59)	637(6.92)	412(14.79)	
High school	2514(39.38)	1680(37.54)	834(46.19)	
Above high school	2556(52.03)	2012(55.54)	544(39.02)	
BMI, kg/m^2^	33.20(0.15)	33.61(0.16)	31.68(0.31)	<0.001
OBS	19.36(0.14)	19.84(0.17)	17.58(0.23)	<0.001
Dietary OBS	15.96(0.14)	16.36(0.16)	14.48(0.21)	<0.001
Lifestyle OBS	3.39(0.031)	3.47(0.04)	3.10(0.04)	<0.001
Drinking status, n (%)				<0.001
No	2840(39.15)	1762(34.65)	1078(55.83)	
Moderate	2539(48.36)	1963(51.27)	576(37.57)	
Heavy	740(12.50)	604(14.09)	136(6.59)	
Smoking status, n (%)				<0.001
Former	2133(35.42)	1372(33.52)	761(42.49)	
Never	2990(48.27)	2270(51.05)	720(37.99)	
Now	996(16.30)	687(15.44)	309(19.52)	
Hypertension, n (%)				<0.001
No	1695(29.25)	1353(32.33)	342(17.84)	
Yes	4424(70.75)	2976(67.67)	1448(82.16)	

PIR, poverty income ratio; BMI, body mass index; OBS, oxidative balance score.

All values are expressed as a weighted proportion (%) or mean ± standard error.

### 3.2 Relationships between OBS, ACM and CVM

During an average follow-up period of 100.9 months, there were 1,790 cases of ACM and 508 cases of CVM. In multivariate Cox regression models (**Table 3**), patients with T2DM in Q4 showed significantly lower risk of ACM and CVM compared with patients in Q1. Specifically, in model 1, patients in Q4 had an HR of 0.47 (95% CI: 0.38-0.58) for ACM and 0.39 (95% CI: 0.25-0.59) for CVM. Even after adjusting for confounders such as age, sex, race, PIR, marital status, education level, smoking status, and hypertension, patients in Q4 had an HR of 0.70 (95% CI: 0.58-0.86) for ACM and 0.57 (95% CI: 0.36-0.88) for CVM.

**Table 3:** Association between OBS and mortality of the T2DM population.

	Model 1		Model 2		Model 3	
	HR (95%CI)	P value	HR (95%CI)	P value	HR (95%CI)	P value
All-cause mortality						
OBS	0.96(0.95,0.97)	<0.001	0.96(0.95,0.97)	<0.001	0.98(0.97,0.99)	<0.001
OBS quartiles						
Q1	ref		ref		ref	
Q2	0.69(0.57,0.84)	<0.001	0.67(0.56,0.82)	<0.001	0.74(0.61,0.89)	0.002
Q3	0.63(0.54,0.75)	<0.001	0.65(0.55,0.77)	<0.001	0.78(0.67,0.92)	0.003
Q4	0.47(0.38,0.58)	<0.001	0.52(0.43,0.63)	<0.001	0.70(0.58,0.86)	<0.001
P for trend		<0.001		<0.001		<0.001
Cardiovascular mortality						
OBS	0.95(0.93,0.97)	<0.001	0.95(0.93,0.97)	<0.001	0.97(0.95,0.99)	0.003
OBS quartiles						
Q1	ref		ref		ref	
Q2	0.66(0.46,0.94)	0.023	0.64(0.45,0.90)	0.011	0.70(0.50,0.98)	0.036
Q3	0.64(0.45,0.91)	0.014	0.65(0.46,0.92)	0.016	0.78(0.56,1.09)	0.140
Q4	0.39(0.25,0.59)	<0.001	0.42(0.27,0.66)	<0.001	0.57(0.36,0.88)	0.011
P for trend		<0.001		<0.001		0.021

Model 1: no adjusted.

Model 2: adjusted for age, sex, and race.

Model 3: further adjusted for PIR, marital status, education level, smoking status, and hypertension based on Model 2.

PIR, poverty income ratio; OBS, oxidative balance score; T2DM, diabetes mellitus; HR, hazard ratio; CI, confidence interval.

**Figure 2:**
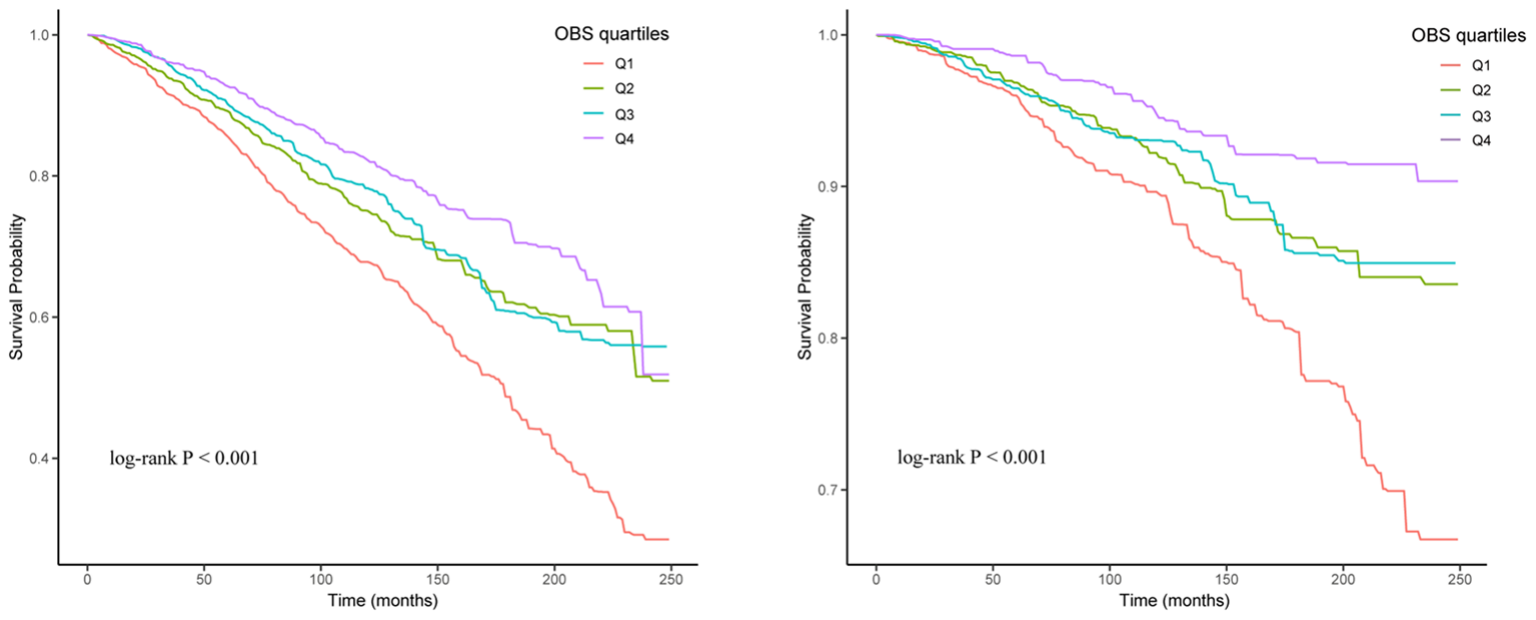
Kaplan-Meier curve of the OBS group for all-cause mortality (Left) and cardiovascular mortality (Right).

**Figure 3:**
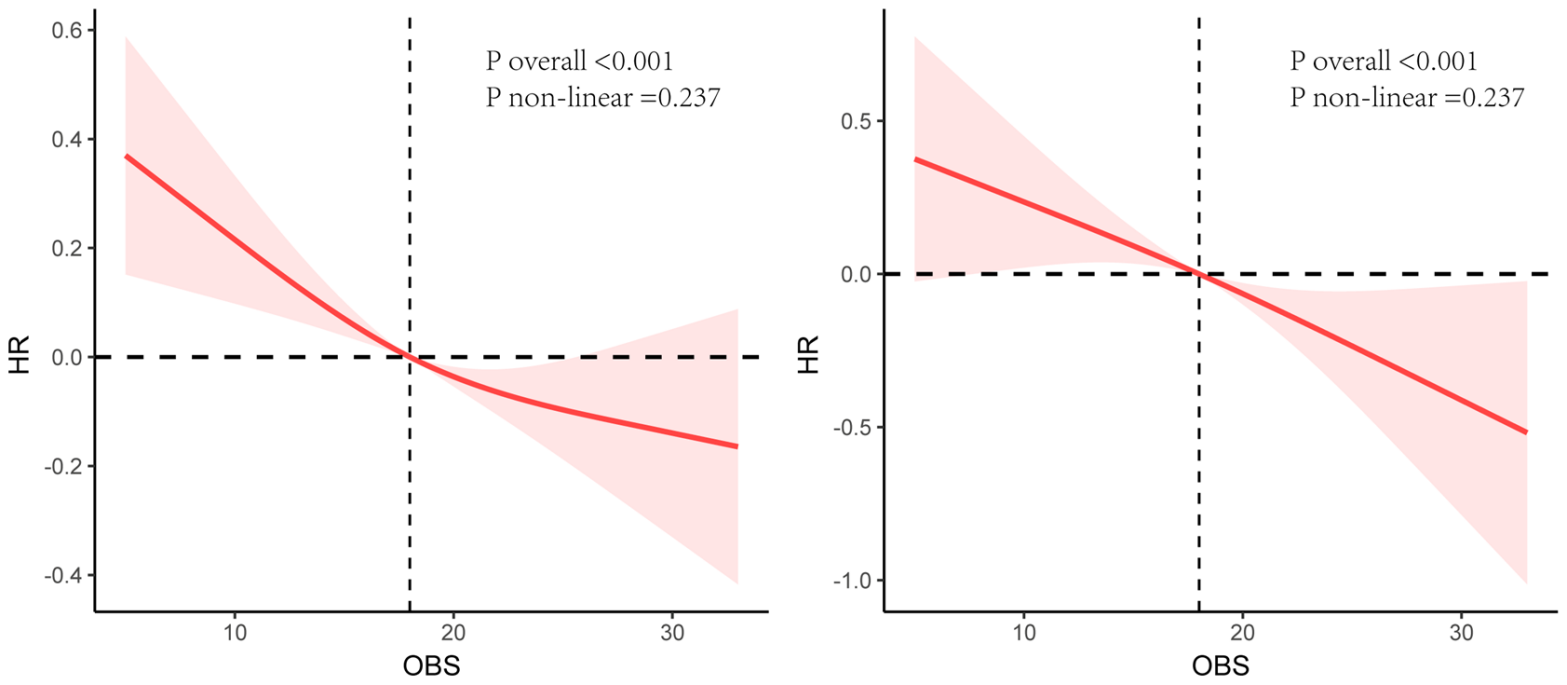
Restricted cubic splines analysis between OBS, all-cause mortality (Left), and cardiovascular mortality (Right).

The Kaplan-Meier curves and the log-rank test revealed significant differences among the four groups, with the Q4 group demonstrating a higher survival probability (**Figure 2**). RCS analysis indicated a linear relationship between OBS, ACM and CVM, with ACM and CVM increasing with higher OBS (**Figure 3**).

### 3.3 Relationships between lifestyle, dietary OBS, ACM, and CVM

**Table 4** illustrates the relationship between lifestyle, dietary OBS, and both ACM and CVM in T2DM. As continuous variables, both dietary OBS and lifestyle OBS were significantly associated with a decreased risk of ACM in T2DM (lifestyle OBS: HR = 0.91, 95% CI 0.87-0.96, P < 0.001; dietary OBS: HR = 0.98, 95% CI 0.97-0.99, P < 0.05) and CVM (lifestyle OBS: HR = 0.89, 95% CI 0.82-0.97, P < 0.05; dietary OBS: HR = 0.97, 95% CI 0.95-0.99, P < 0.05) in fully adjusted Model 3.

**Table 4:** Association between lifestyle OBS, dietary OBS, and mortality of T2DM.

	Model 1		Model 2		Model 3	
	HR (95%CI)	P value	HR (95%CI)	P value	HR (95%CI)	P value
All-cause mortality						
Lifestyle OBS	0.92(0.88,0.97)	0.001	0.84(0.80,0.88)	<0.001	0.91(0.87,0.96)	<0.001
Dietary OBS	0.96(0.95,0.97)	<0.001	0.97(0.96,0.98)	<0.001	0.98(0.97,0.99)	0.002
Cardiovascular mortality						
Lifestyle OBS	0.91(0.85,0.98)	0.016	0.82(0.76,0.89)	<0.001	0.89(0.82,0.97)	0.008
Dietary OBS	0.95(0.93,0.97)	<0.001	0.96(0.94,0.98)	<0.001	0.97(0.95,0.99)	0.009

Model 1: no adjusted.

Model 2: adjusted for age, sex, and race.

Model 3: further adjusted for PIR, marital status, education level, smoking status, and hypertension based on Model 2.

PIR, poverty income ratio; OBS, oxidative balance score; T2DM, diabetes mellitus; HR, hazard ratio; CI, confidence interval.

### 3.4 Subgroup analysis

In patients with T2DM, those with high OBS consistently demonstrate lower risks of ACM and CVM across different subgroups based on age, sex, BMI, smoking, and hypertension, as shown in **Tables 5**, **6**. The interaction showed that the presence of hypertensive disorders influenced the negative association between OBS and CVM.

**Table 5:** Subgroup analysis between OBS and ACM in T2DM population.

	HR (95% CI)	P value	P for interaction
Age, years			0.607
≥60	0.98(0.96,0.99)	<0.001	
<60	0.99(0.97,1.01)	0.210	
Sex			0.924
male	0.98(0.97,0.99)	0.003	
female	0.98(0.96,1.00)	0.013	
BMI			0.220
<30	0.98(0.97,1.00)	0.024	
≥30	0.97(0.96,0.99)	<0.001	
Smoking			0.953
former	0.98(0.96,0.99)	0.009	
now	0.97(0.95,1.00)	0.016	
never	0.98(0.97,1.00)	0.015	
Hypertension			0.071
yes	0.98(0.97,0.99)	0.003	
no	0.96(0.94,0.99)	0.002	

**Table 6:** Subgroup analysis between OBS and CVM in the T2DM population.

	HR (95% CI)	P value	P for interaction
Age, years			0.169
≥60	0.97(0.95,0.99)	0.005	
<60	0.96(0.91,1.00)	0.044	
Sex			0.518
male	0.96(0.94,0.98)	0.001	
female	0.97(0.95,1.00)	0.062	
BMI			0.160
<30	0.98(0.95,1.00)	0.101	
≥30	0.96(0.93,0.98)	0.002	
Smoking			0.173
former	0.96(0.93,0.99)	0.019	
now	0.93(0.89,0.97)	<0.001	
never	0.99(0.96,1.01)	0.325	
Hypertension			0.016
yes	0.98(0.95,1.00)	0.058	
no	0.93(0.90,0.96)	<0.001	

## 4 Discussion

In this large-scale retrospective study, we identified a significant negative correlation between OBS and both ACM and CVM among T2DM patients, even after adjusting for confounding factors such as age, sex, BMI, smoking, and hypertension. Our findings suggest that higher OBS is associated with a reduced risk of ACM and CVM. These results underscore the importance of promoting health-conscious behaviors, particularly in dietary and lifestyle OBS adjustment, among individuals with T2DM, potentially decreasing ACM and CVM.

The two main features of T2DM are insulin resistance in target tissues and a relative deficiency in insulin production by pancreatic β-cells, and the production of reactive oxygen species (ROS) is closely related to insulin resistance (23). The ROS production and the antioxidant defense system imbalance lead to oxidative stress (OS). The antioxidant defense system can also reduce ROS accumulation, alleviating oxidative stress (24). The interplay between pro-oxidant and antioxidant factors determines an individual’s oxidative balance. Van Hoydonck et al. first introduced the concept of the OBS, which comprehensively assesses this state by considering dietary intake of vitamin C, beta-carotene, and iron (25).

OBS is now widely used in epidemiological research to assess the association between OS and the risk of chronic diseases (26). Studies have found that OBS is variably associated with reduced risks of T2DM, cardiovascular diseases, chronic kidney disease, periodontitis, sleep disorders, colorectal adenomas, and colorectal cancer (8, 9, 13, 14, 27–29). OBS is also helpful in predicting clinical outcomes and is significantly negatively correlated with all-cause, cardiovascular, and cancer mortality (16, 26). Moreover, current research indicates that OBS can serve as a valuable predictor of prognosis. Early identification of high-risk T2DM patients is crucial for improving prognosis. Existing studies suggest a U-shaped association between the triglyceride-glucose (TyG) index and all-cause as well as CVM in US individuals with diabetes or prediabetes (30). Another study indicates that the TyG-BMI index in US elderly diabetic patients is U-shapedly associated with ACM and linearly associated with CVM (31). Physiologically, OBS may influence health outcomes by modulating the state of OS in the body. OS is thought to be one of the key factors in the development of diabetes and its complications because it can damage cellular components, including lipids, proteins, and nucleic acids (32).

Kwon et al. conducted a study involving 7,369 participants aged 40-69 years enrolled in the Korean Genome and Epidemiology Study (33). They found that during a mean follow-up period of 13.6 years, 908 men and 880 women developed T2DM. The conclusion drawn was that individual with high OBS had a lower risk of developing T2DM. Previous studies have suggested the potential role of OBS in the risk of ACM and CVM. Rodriguez et al. conducted a prospective investigation of participants in the Seguimiento Universidad de Navarra Study and found a negative correlation between OBS, ACM and CVM (16). However, this study only focused on university graduates aged 20 and above. The study by Hoydonck et al. targeted male smokers; Kong et al. focused on individuals at high risk of cardiovascular disease; and Mao et al. conducted a similar study among older women in Iowa (25, 34, 35). However, these studies did not consider the relationship between OS and T2DM, nor did they account for the impact of OBS on this specific population. Utilizing the NHANES database, our study benefits from a large amount of observational data and long-term follow-up, taking full advantage of its inclusion of diverse racial backgrounds, educational levels, etc., thus contributing to the current body of research evidence.

In our results, the increase in PIR and education level from Q1 to Q4 in participants is consistent with previous studies, where individuals who are better off or have a higher level of education are more likely to adopt health-conscious behaviors, including a higher intake of antioxidants, which can help to improve their oxidative homeostasis and thus reduce the risk of ACM and CVM (36, 37). In addition, the interaction showed that the presence or absence of comorbid hypertensive disease in patients with T2DM affected the negative association between OBS and CVD. Previous studies have identified that hypertensive disease increases the body’s OS, inflammatory response, and vasoconstriction, and leads to structural changes in the heart and blood vessels and that these changes lead to an increased risk of cardiovascular mortality in patients with T2DM, thereby interfering with OBS (38, 39).

Our study possesses several strengths. Firstly, utilizing the nationally representative NHANES population constitutes the primary and most significant advantage. Secondly, subgroup analysis enhances the robustness of the observed relationship between OBS, ACM, and CVM in T2DM patients, underscoring the reliability of the findings regardless of other cardiovascular risk factors such as age, sex, BMI, smoking, and hypertension. Furthermore, we conducted independent investigations into the influence of lifestyle OBS and dietary OBS on the association between ACM and CVM in T2DM patients.

Our study also exhibits several limitations. Firstly, OBS was only measured at baseline, thereby precluding consideration of potential changes or fluctuations in OBS over time. Consequently, our analysis cannot address longitudinal variations in OBS. To overcome the limitation of measuring OBS only at baseline, future studies may consider prospective cohort studies in which OBS is measured at regular intervals to assess its changes over time and its long-term impact on health outcomes. Secondly, our findings cannot establish causal relationships due to the inherent nature of observational study designs. Moreover, given the intricate interplay of factors influencing the association between T2DM and all-cause and CVM, we endeavored to incorporate numerous covariates, encompassing age, sex, race, PIR, marital status, education level, smoking status, and hypertension. Nevertheless, despite adjusting for these variables, residual confounding factors may persist. Finally, this is a population-based study conducted among T2DM patients in the US. Although it includes 1,198 Mexican Americans, 1,522 Non-Hispanic Black individuals, 2,452 Non-Hispanic White individuals, 526 Other Hispanic individuals, and 421 individuals of other races, our study results may not be generalizable to other populations.

## 5 Conclusion

For the first time, we revealed an association between OBS, ACM, and CVM among patients with T2DM. We demonstrated that there was a linear association between OBS, ACM, and CVM.

## Data Availability

The original contributions presented in the study are included in the article/supplementary material. Further inquiries can be directed to the corresponding authors.
